# From novice to expert: the impact of peer review interaction on authors’ academic growth

**DOI:** 10.3389/fpsyg.2025.1642718

**Published:** 2026-01-12

**Authors:** Xuehua Wang, Meihua Chen

**Affiliations:** School of Foreign Languages, Southeast University, Nanjing, China

**Keywords:** academic growth, academic writing, novice and expert authors, occluded genres, peer review interaction

## Abstract

As a core link in academic quality control, peer review has been proven to improve the quality of papers in paper publishing, but this is often limited to the micro level of individual papers, the macro-level impact of peer review on authors’ academic development is not yet clear. This study adopts a longitudinal case study to analyze the publication process of two international journal papers with a 14 year interval by the same author. Combining review comments, author responses, and interview reflections, the study systematically examines the development trajectory of three dimensions: paper quality, social skills and academic participation. Research has found that peer review interaction drives deep transformation through a triple mechanism: the internalization of paper standards sparks a shift from mechanical imitation to intentional construction, the practice of responding to comments promotes a shift from unidirectional acceptance to negotiated revision, and the change in academic participation undergoes a shift from external regulation to intrinsic motivation. The research results provide an empirical basis for academic writing teaching and a dynamic analysis for the study of occluded genres.

## 1 Introduction

Since Swales’ (1990) defined the concept of “genre,” the discourse structure and rhetorical features of academic genres, such as research articles, have formed a complete analytical paradigm (Hyland, 2016). However, peer review reports, as “occluded genres” of academic publishing system due to their non-public nature have long been on the edge of research (Paltridge, 2017). Such research imbalance is particularly significant in the EAP (English for Academic Purposes) field: although most of SCI and SSCI journals adopt a peer review system (Bornmann, 2011), review interaction is not included in the most frequently explored research topics (Yang et al., 2023; Hyland and Jiang, 2021).

Despite this, researchers have confirmed the crucial role of peer review in maintaining academic standards through this quality filtering mechanism (Bornmann and Daniel, 2008). Empirical researches have shown that review comments can significantly enhance the methodological rigor (Jefferson et al., 2007) and clarity of arguments (Matsuda and Tardy, 2007) of papers, but this improvement is often limited to the micro level of individual papers. From the perspective of academic socialization theory (Lave and Wenger, 1991), the repeated critical dialogue (Hyland, 2015) during the review process may contain deeper cognitive restructuring mechanisms (Tardy, 2016): novice authors gradually internalize the evaluation criteria of the discipline discourse community and construct their academic competences by interacting with reviewers through comments and responses (Tardy, 2009; Flowerdew, 2015).

Therefore, this research is aimed to track the long-term effects of review interaction, adopting a longitudinal case study to track paper writing, review, and revision trajectory of one author spanning over 10 years, exploring:

1. The changes in paper quality at novice and expert stages.
2. The changes in academic social skills at novice and expert stages.
3. The changes in academic participation at novice and expert stages.

The results of this research will provide empirical evidence for the teaching of academic writing and open up a dynamic analysis path for the research of occluded genres.

## 2 Literature review

### 2.1 Peer review and paper quality

Peer review is a core mechanism in academic publishing to ensure the quality of papers. By providing feedback and improvement suggestions, it significantly enhances the academic level and readability of papers (Hyland, 2016). Researches have shown that review comments can help authors identify weak areas in their research, such as deficiencies in method design, inadequate data analysis, and unclear writing expression (e.g., Paltridge and Starfield, 2016; Swales and Feak, 2012). The types of review comments have a particularly significant impact on the quality of the paper: comments on research methods can often enhance the rigor of the research; comments on data can help improve the reliability of results; comments on writing can improve the logic and readability of the paper (Flowerdew, 2015). In addition, effective review interaction can also stimulate authors’ critical thinking, prompting them to examine their research from multiple perspectives, thereby further improving the quality of the paper (Tardy, 2012; Wu et al., 2023).

### 2.2 Peer review and academic socialization

Peer review is also an important mechanism for academic socialization of authors (Curry and Lillis, 2013). Through review feedback, the author is able to gradually master the norms of academic writing, the application of research methods, and the accumulation of discipline knowledge (Tardy, 2006). For novice authors, review comments are particularly important as they provide valuable guidance to help them understand the expectations and standards of the disciplinary discourse community (Swales’, 1990). In addition, interactive review can cultivate the author’s critical thinking and problem-solving abilities, making them more independent and confident in future research (Belcher, 1994). The academic growth from novice to expert is a complex social construction process (Lave and Wenger, 1991; Cowley, 2015). Social constructivist theory holds that knowledge is constructed through social interaction, and peer review, as an important form of academic interaction, provides authors with opportunities for learning and growth (Engeström, 2001; Sandewall, 2012), which provides an important entry point for the current research.

## 3 Materials and methods

### 3.1 Data collection

This research used the purposeful sampling (Creswell and Poth, 2018; Okojie et al., 2022) to select research subject based on the following criteria: Firstly, the target author should have been engaged in academic research in the same field for at least 10 years, which ensures that academic trajectory can fully cover the critical transition period from being a novice to an expert author (Flowerdew, 2015). Secondly, the journal review system in the author’s disciplinary field is well-established and highly standardized, and the review comments usually include detailed technical feedback (Bazerman, 1988). What’s more, the required data are accessible: documents of chosen papers including first submitted manuscripts, multiple rounds of review comments and responses, editorial decision letters, and resubmitted and final manuscripts, and semi-structured in-depth interviews to trace the evolution of author’s response strategies and academic participation. This dual data requirement can cross validate text revision traces and author reflective narratives, revealing the academic growth in occluded genre interactions.

Thus, we focused on a university faculty member in the field of electrical and electronics engineering, by the name of Song (pseudonym) with SCI (Science Citation Index) publishing experiences of more than 10 years. We chose the field of electrical and electronic field lies in its rapid technological iteration and close interdisciplinary collaboration. These characteristics require the peer review process to handle more complex technical disputes, which provides typical scenarios for observing how novice authors absorb disciplinary conventions and establish technical judgment through interactive review. Then two of his first-author papers (Paper 2007 and Paper 2021) were selected for the following reasons. (1) The journals of the two papers are IEEE (Institute of Electrical and Electronics Engineers) transactions with impact factors of 4.993 and 5.6, respectively, employing a rigorously standardized peer-review system. We use ITX and ITY to represent these two transactions, respectively. (2) The two papers were published 14 years apart. Paper 2007 was Song’s first SCI paper in his academic life. At that time, he was an associate professor and had previously published papers in domestic journals and domestic English journals. Later, Song was promoted to professor and became doctoral supervisor. Paper 2021 is one of his recent first-author papers after publishing over 60 SCI papers with more than 1700 citations. Therefore, these two papers are believed to respectively represent the novice and expert stages of Song’s SCI paper publishing. (3) The documents of the two papers are complete. Apart from final version, the first submitted, resubmitted versions of the two papers and the complete correspondences between Song and the two transactions, i.e., correspondences between Song and the editors and reviewers were collected. Based on these correspondences, we listed the timeline of the publication process of the two papers (see Table 1).

**TABLE 1:** A timeline of the publication process of Song’s two papers.

Paper 2007		Paper 2021	
25-Jul-2005	The first submission to ITX	16-Jan-2020	The first submission to ITY
31-Oct-2005	Editor’s decision letter of major revision	19-Feb-2020	Editor’s decision letter of major revision
08-Dec-2005	The first re-submission to ITX	10-May-2020	The first re-submission to ITY
13-Mar-2006	Editor’s decision letter of being accepted with mandatory revision.	08-Jun-2020	Editor’s decision letter of minor revision
29-Mar-2006	The second re-submission to ITX	16-Jul-2020	The second re-submission to ITY
05-Apr-2006	Editor’s decision letter of being accepted	26-Sep-2020	Editor’s decision letter of being accepted
Mar 2007	Publication in ITX	Oct 2021	Publication in ITY

Three interviews with Song were conducted, the themes of which were summarized in Table 2. Each of the interview lasted about 30 min, and all were recorded with permission. Following each interview, notes were summarized and those parts of the interviews that seemed to be particularly relevant and illustrative were transcribed. Furthermore, inquires to clarify specific questions were made through email contact with Song.

**TABLE 2:** Themes of the three interviews with Song.

Time	Point of time during the current study	Theme of the interview
9 Mar 2024	After deciding to conduct the current research	Song’s academic background, attitude toward international publication, and paper selection based on our research aim.
20 May 2024	After studying original and revised versions of the two papers, and reviewers’ comments and author’s responses of the papers	Song’s reflection on the publication processes of the two papers and comments them.
29 Oct 2024	During the period of drafting the manuscript	The impact of the publication on Song’s academic career and his current view toward international publishing

This study strictly adheres to ethical standards, all data is anonymized, and the faculty member’ informed consent is obtained.

### 3.2 Data analysis

This study adopts a mixed research method, combining text analysis, interaction analysis, and interview analysis, to comprehensively explore the impact of peer review interaction on Song’s academic growth.

#### 3.2.1 Text analysis

Text analysis is mainly based on the review comments from reviewers. The statistics of the number of review comments in two rounds of two papers are shown in Table 3. One thing to note is that one reviewer was added in the final round of review of Paper 2021.

**TABLE 3:** Number of comments from reviewers of the two papers.

Phase	Paper 2007 (3 reviewers)	Paper 2021 (2 + 1 reviewers)
1st round of review	20 comments	18 comments
2nd round of review	15 comments	5 comments
Being accepted	0 comments	9 comments from an added reviewer (reviewer 3)

In the light of Halliday’s (1985) theory of systemic functional linguistics, a three-dimensional analysis framework was constructed according to different focuses, including technical depth, scholarly engagement, and text organization (see Table 4).

**TABLE 4:** Three-dimensional analysis framework of reviewers’ comments.

Three meta-functions of language	Types of reviewers’ comments
Ideational function	Technical depth
Interpersonal function	Scholarly engagement
Textual function	Text organization

##### 3.2.1.1 Technical depth dimension (ideational function)

It involves technical issues such as algorithm improvement, experimental design, and theoretical analysis. For example: it was pointed out that the paper lacks performance comparison data:

The major concern of this reviewer is the lack of comparative results. The authors merely say that. For a precise analysis, this reviewer would have expected at least a comparison with … or with … (which is missing in the figures) (Comment 1, Paper 2007)

##### 3.2.1.2 Scholarly engagement dimension (interpersonal function)

It involves literature citations, comparison with existing researches, and clarity of academic contributions. For example: it was pointed out that the introduction section should clearly state the original contribution:

The introduction should be better organized, putting in evidence the original contributions of the paper. Only few lines (in the last but one paragraph of page 3) are dedicated to the paper content. (Comment 6, Paper 2007)

##### 3.2.1.3 Text organization dimension (textual function)

It involves paper structure, clarity of figures and tables, use of terminology, formula errors, etc. For example, a formula error was pointed out:

Eq. (6): there is a “j” missing before 2p. (Comment 4, Paper 2007)

Based on this framework, the 38 review comments in the first round review of two papers were first classified, and this work was independently conducted by two researchers in this paper. The study mainly focuses on the first round of review and response, based on the following three reasons: Firstly, the initial review is a key turning point in the author’s paper writing practice, during which the intensity of cognitive reconstruction is most significant; Secondly, the first round of interaction has methodological purity, which can effectively control the cumulative effects and other factors such as editorial arbitration generated in subsequent rounds.

The inter rater reliability were evaluated using Cohen’s Kappa coefficient and the result was 0.810, which reached the level of substantial consistency according to Landis and Koch’s (1977) criteria. The percentage consistency matrix (see Table 5) further shows that the consistency of the text organization dimension is the highest (95.5%), followed by technical depth (83.3%), while the consistency of the scholarly engagement dimension is the lowest (75.0%), mainly with technical depth dimension. This result confirms the high reliability of the classification framework.

**TABLE 5:** Percentage consistency matrix of three dimensions of reviewers’ comments.

Category	Researcher B: technical depth	Researcher B: scholarly engagement	Researcher B: text organization
Researcher A: technical depth	83.3% (10/12)	8.3% (1/12)	8.3% (1/12)
Researcher A: scholarly engagement	25.0% (1/4)	75.0% (3/4)	0.0% (0/4)
Researcher A: text organization	4.5% (1/22)	0.0% (0/22)	95.5% (21/22)

For the disagreement in the classification, the two researchers reviewed the review comments and classification criteria, and consulted with the paper author if necessary, to reach an agreement after discussion: Here is one of the examples:

The algorithm is rather old with respect to recent algorithms such as [X] and [Y], which are based on an efficient use of SAR processing algorithms for … mode because they fully compensate the… of targets through … (Comment 12, Paper 2007)

Two researchers initially labeled scholarly engagement and technology depth separately, but after discussion and confirmation with the paper author, they unanimously agreed that although the algorithm was mentioned in the comments, it actually pointed out that the algorithm may be outdated and its true intention is to inquire about the innovation and academic contribution of this study in the context. Therefore, it was ultimately classified as scholarly interaction.

Then, quantitative statistics and comparative analysis were conducted on the 38 review comments of two papers to reveal the change of paper quality of the same author at different stages.

#### 3.2.2 Interaction analysis

Based on Swales’ (2004) theory of academic discourse interaction, this research examined the comments and responses of two papers, and divided the interaction modes into three categories: unidirectional acceptance, negotiated revision, and position maintenance.

##### 3.2.2.1 Unidirectional acceptance

This is manifested as the author fully adopting the review suggestions without raising any objections or explanations, and their responses often use programmatic acknowledgements. For example, it was pointed out an error in description of formula: “Page 4, line 24: “dividing the recurrent of… by N” should be “dividing the sampling frequency by N”” (Comment 3, Paper 2007). Author responded:

Thank you for your suggestion, we have made this change.

##### 3.2.2.2 Negotiated revision

In this mode, the author adds explanatory notes when partially adopting suggestions, generally using transitional words such as “however” to engage in rational dialogue and present a limited stance maintenance. For example, “In abstract and Section I, the authors say that subaperture processing is not appropriate for. I doubt it, in my opinion, the subaperture method has no obvious flaws, …, so suggest don’t add subaperture part.” (Comment 3, Paper 2021). Author responded:

We agree with the reviewer that the subaperture method has no obvious flaws, and we also use it very extensively. In response to the reviewer’s suggestion, we have eliminated the comments on…, which are highlighted in the revised manuscript.
However, we believe that the full-aperture technique presented in our paper could also have some merits. …Therefore, we still believe the significance of the full-aperture processing technique, at least from the viewpoint of academic research.

##### 3.2.2.3 Position maintenance

This is manifested as the author using theoretical deduction or experimental data to demonstrate the rationality of the original viewpoint, politely refusing to modify and presenting a stance to maintain. For example, reviewer questioned the improper use of terminology: “It is only the change of the P phase to Q phase, should not be called as “AB.” You don’t have to make up a word” (Comment 2, Paper 2007). Author responded:

We are very sorry to make such confusion here. As the reviewer has indicated, the “AB” is indeed the change of the P phase to Q phase. However, we call it AB just following the classical paper by…

Based on this category, the 38 responses to the first round comments have been classified, and this work was independently conducted by two researchers. The inter rater reliability were evaluated using Cohen’s Kappa coefficient and the result was 0.718, which reached the level of substantial consistency according to Landis and Koch’s (1977) criteria. The percentage consistency matrix (see Table 6) further shows that the consistency of “position maintenance” mode achieved perfect consistency (100.0%), followed by “unidirectional acceptance” (88.9.3%), while “negotiated revision” mode showed relatively lower agreement (60.0%). This result confirms the high reliability of the classification framework and suggests that the criteria for defining “negotiated revision” may be further modified.

**TABLE 6:** Percentage consistency matrix of three author’s interact modes.

Category	Researcher B: unidirectional acceptance	Researcher B: negotiated revision	Researcher B: position maintenance
Researcher A: unidirectional acceptance	88.9% (24/27)	11.1% (3/27)	0.0% (0/27)
Researcher A: negotiated revision	40.0% (2/5)	60.0% (3/5)	0.0% (0/5)
Researcher A: position maintenance	0.0% (0/6)	0.0% (0/6)	100.0% (6/6)

For the disagreement in the classification, the two researchers reviewed the review comments and responses, and consulted with the paper author if necessary, to reach an agreement after discussion. Here is one of the examples:

Page …, line …: It should be stated here that, for the classical azimuth scaling algorithm, the … process will satisfy Eq. (36) and (37). (comment 13, Paper 2021)

This is a review comment classified as technical depth category, and two researchers have classified the response to the comment as “unidirectional acceptance” and “negotiated revision” respectively based on the author’s response below:

Thank you for your reminding. We have emphasized it in the revised manuscript that in the framework of…, azimuth scaling induces, and when this happens Eq. (36) < (37). However, Eq. (36) and (37) are aiming at the full-aperture signal after processed by…. For subaperture processing, since … is not used, Eq. (36) and (37) are not applicable. Nevertheless, for subaperture processing, it is a quite clear and well known fact that azimuth scaling does not give rise to any ….

This response acknowledged the reviewer’s comments and mentioned that the problem has been emphasized in the manuscript. However, the author then pointed out that for the sub aperture processing mentioned in the comment, Eq. (36) and (37) are not applicable as Eq. (36) and (37) are aiming at the full aperture signal. Therefore, it was ultimately classified as “negotiated revision.”

Then, quantitative statistics and comparative analysis were conducted on the 38 responses of the two papers to reveal the strategy changes of academic social skills of the same author at different stages.

### 3.3 Interview analysis

The interview data analysis adopted the thematic analysis framework (Braun and Clarke, 2006; Alves de Castro, 2023). Firstly, the researchers repeatedly read the transcribed text and encoded sentences one by one to generate initial labels. Then, by continuously comparing and categorizing, the sub themes and core themes are ultimately generated. To ensure reliability, the coding process was independently completed by two researchers. Any disagreements were then resolved through discussion to reach a final consensus.

Based on the mixed research method proposed in this research, the interview data will also be presented in the results of first two research questions.

## 4 Results

### 4.1 The changes in paper quality at different stages

Next, we will examine the changes in paper quality at novice and expert stages from the perspectives of review comments and interview reflection

#### 4.1.1 Perspectives of review comments

The first round of decision letter for both Paper 2007 and Paper 2021 were major revisions. Each reviewer (3 reviewers from Paper 2007, 2 reviewers from Paper 2021) made an opening remark on the paper before providing specific review comments:

The paper describes the application of. The idea, already used for…, is extended to the range dimension. The mathematical details are almost always clear. Nevertheless, the major concern of this reviewer is the lack of comparative results… (Reviewer 1, Paper 2007)
The paper presents a method for. The authors present a simple extension of well-known algorithms to. However, the introduction should be better organized, putting in evidence the original contribution of the paper… (Reviewer 2, Paper 2007)
The manuscript discusses the application of … The idea is simple, but I have three main concerns about its publication … (Reviewer 3, Paper 2007)
First, let me explain my understanding of this article. In my opinion, the method in this paper can be regarded as two convolution operations…ITY is not recommended if the method is the same as I described. (Reviewer 1, Paper 2021)
This paper describes an extended two step approach to. The two step approach is useful in obtaining …. Therefore, in my opinion, the manuscript is dealing with a very essential aspect of … that is certainly worth investigating. Nevertheless, there are still some changes that must be applied. (Reviewer 2, Paper 2021)

We found that the three opening remarks of Paper 2007 were consistent: the new algorithm proposed in the paper were regarded as a simple extension of the classical algorithm, but the paper needed major revisions. However, the two opening remarks of Paper2021 were contradictory, reviewer 1 advocated that it was not recommended for publication in ITY while reviewer 2 acknowledged the contribution of the paper. The different opinions of the two reviewers probably explained why the editor made a major revision decision for this expert-stage paper and why the editor invited another reviewer to review the paper before final acceptance.

Next, we will focus on the detailed comments. The first round of review for paper 2007 and paper 2021 had a total of 20 and 18 specific review comments, respectively. Based on the three-dimensional analysis framework of technical depth, scholarly engagement and text organization, we generated the quantitative statistics in Table 7.

**TABLE 7:** Comparative analysis of review comment classifications: Paper 2007 vs. Paper 2021.

Category	Paper 2007	Paper 2021	Rank (2007)	Rank (2021)	Difference (2021–2007)	95% Confidence interval for difference
Technical depth	7 (35%)	7 (39%)	2	2	+4%	[−27.1%, +35.1%]
Scholarly engagement	4 (20%)	3 (17%)	3	3	−3%	[−23.0%, +17.0%]
Text organization	9 (45%)	8 (44%)	1	1	−1%	[−28.7%, +26.7%}
Total	20	18				

Statistical notes: Rank: Categories are ranked (1 = highest, 3 = lowest) based on their percentage frequency within each paper. The rank order remained entirely stable between two papers. Confidence intervals: 95% confidence intervals for the difference in independent proportions were calculated using the method of Agresti and Caffo (2000). All intervals contain zero, indicating that the observed differences are not statistically significant at the α = 0.05 level.

From the distribution data of two papers, respectively, the focus of reviewers shows a stable characteristic. Although the proportion of comments on technical depth increased slightly from 35% to 39% (Difference:+4%), scholarly engagement decreased slightly from 20% to 17% (Difference: −3%), and text organization maintained a dominant position (44%–45%; Difference: −1%), none of these changes were statistically significant. This is evidenced by the chi square test results (χ^2^ = 0.096, p≈0.95) and further supported by the 95% confidence intervals for the differences in proportions, all of which contain zero (see Table 5). This overall stability reflects a consistent review framework in IEEE transactions. Among the three dimensions, the consistent dominance of text organization may be related to the fact that text organization problems are readily identifiable in a short review time, whereas assessing technical depth and scholarly engagement problems may require deeper, field-specific expertise.

#### 4.1.2 Perspectives of interview reflection

Now let’s take a look at the author’s reflection on the submission of the two papers. Paper 2007 is Song’s first SCI paper. Song developed an efficient algorithm to the problem of SAR (Synthetic aperture radar) image formation based on over 1 year of efforts. With this new algorithm and simulation data, he decided to write a paper and submitted to ITX, a renowned IEEE transaction with high impact factor.

At that time I just wanted to make it published internationally without considering much about what a high-quality paper should look like. I just mechanically wrote it based on my own understanding of IEEE SCI papers. (Interview, May 2024)

Three months after submitting, Song received the decision from the editor as “revised and resubmitted (RQ).^1^ “Song’s first reaction was that the paper was rejected, which was also expected. However, after further checking, he found out that this decision was actually provisionally accepted. He was overjoyed and decided to made serious and careful revisions.

After reading the reviewer’s comments for the first time, I suddenly gained a new understanding of what kind of paper can be considered an SCI paper. For example, in terms of structure, the submission version follows the basic structure of introduction, method, results, and conclusion. The introduction presents the problem and the contribution of this paper, the method provides a detailed description of the new algorithm, followed by simulation results, and finally the conclusion summarizes the solution of the problem. However, for each specific paper, mechanically completing each section in isolation is far from enough. It actually requires authors to provide a comprehensive explanation of the problem and its solution from multiple perspectives. (Interview, May 2024)

The extended response to review comments refers to the author’s spontaneous modifications to other parts of the paper based on reviewer’s comments. We found at least 4 out of 20 responses from Song revealed extended responses, of which 3 are responses of technical depth and 1 is response of scholarly engagement. The extended responses not only reflect the need to improve the paper quality, but also reflect the author’s growth of internalizing and implementing high-quality paper standards (Mulligan et al., 2013). Song used statements such as “this comment strongly reminds the authors” to inform the reviewer of any other occluded adjustments he made. For example:

… However, this comment strongly reminds the authors to include … in the revised paper, which takes … into account, and nearly changes all the equations in the paper (Comment 2, Paper 2007).
According to the reviewer’s reminding, we have added this point to that paragraph. Moreover, a new section is added to the paper for. (Comment 14, Paper 2007)

It can be seen that the review comments have prompted novice author to deeply consider the requirements of international journal papers and make major adjustments to the entire manuscript, as mentioned by Song in his response to the editor:

In general, the paper is reorganized, where two new sections concerning … are supplied. (Response letter to editor, Paper 2007)

In this round of revision, the length of the paper has been expanded from 22 double spaced pages to 34 pages. The number of figures has increased from 5 to 10, and the number of formulas has increased from 21 to 40. And references have expanded from 8 to 15. In addition to following the reviewer’s comments, two new sections have been added. One concerns the application of the new algorithm in another system, and the other illustrates the application of the new algorithm in two systems with graphs. Song believes that:

The adding of these two sections not only covers the applications of the new algorithm in the two most commonly-used main systems but also provides graphic explanation of the new algorithm, which is a common method of illustration in our field. Thus the application scenarios and effects of the new algorithm become more comprehensive. It was the review comments that activated my understanding of paper construction and how to implement them in a specific paper. (Interview, May 2024)

For novice authors, although they have read a large number of SCI papers, their understanding of paper quality standards is not profound without starting to write. Through their comments, reviewers translate abstract standards into concrete suggestions. This process provides novice authors with valuable insights and fosters a deeper understanding of high-quality papers.

Gradually, Song’s submitted papers were generally accepted after minor revisions, but there are exceptions, such as Paper 2021.

Different from Paper 2007, the extended responses have not been found in Paper 2021. The proposed approach in Paper 2021 was originally an idea conceived of in 2012, which has since been gradually improved and developed in practice. In 2016, the second step of the approach was added, which improved the application of the approach in different scenarios and conditions. Subsequently, experiments were conducted and life experimental data was obtained. After seeing the significant performance results, Song and his research team carried out a rigorous formula derivation and added the process of approach formation. The final paper includes proposal of the problem, a detailed introduction to the two-step new approach, life experimental data analysis, and conclusions. In Song’s words:

This paper has a complete framework in both theory and practice. Two steps of the approach solve problems under different conditions, each with its own innovative point. It is even possible to split the two-step approach into two papers (Interview, May 2024).

The construction of this paper stemmed from Song’ years of experience in submitting and revising papers. In addition, his experiences of being reviewers also helped to have a clear and deep understanding and implementation of quality standards of SCI papers.

### 4.2 The changes in academic social skills at different stages

Now, we will examine the changes in academic social skills at novice and expert stages from Song’s responses to comments in the first round of the two papers.

#### 4.2.1 Mainly relying on unidirectional acceptance at novice stage

We categorized the 38 author responses (20 from Paper 2007, 18 from Paper 2021) into three categories (unidirectional acceptance, negotiated revision and position maintenance). The analysis of author responses reveals a marked shift in strategies between the novice and expert stages (see Table 8). At novice stage, unidirectional acceptance accounted for the highest proportion, reaching 70% (14/20). Among these, all text organization responses were accepted (9/9), most scholarly engagement responses were accepted (3/4). This reflects the strong demand to improve the manuscript and a deep respect for the role of reviewers as academic judges. Conversely, negotiated revision was rare (5%, 1/20), indicating limited experiences in academic negotiation (Li, 2006), leading to a passive cycle of acceptance and revision.

**TABLE 8:** Comparative analysis of review comment responses: Paper 2007 vs. Paper 2021.

Category	Technical depth	Scholarly engagement	Text organization	Total (Percentage)	Rank	Difference (2021–2007)	95% Confidence interval for difference
**Paper 2007**							
Unidirectional acceptance	2	3	9	14 (70%)	1		
Negotiated revision	1			1 (5%)	3		
Position maintenance	4	1		5 (25%)	2		
Total (percentage)	7 (35%)	4 (20%)	9 (45%)	20			
**Paper 2021**							
Unidirectional acceptance	2	1	4	7 (39%)	2	−31%	[−59.4, −0.8%]
Negotiated revision	4		3	7 (39%)	1	+34%	[+7.6%, +57.5%]
Position maintenance	1	2	1	4 (22%)	3	−3%	[−23.0%, +17.0%]
Total (percentage)	7 (39%)	3 (17%)	8 (44%)	18			

Statistical notes: Rank: Categories are ranked (1 = most frequent, 3 = least frequent) based on their total percentage within each paper. The rank order is different between two paper, indicating the change of author’s responses to review comments. Confidence Intervals: 95% confidence intervals for the difference in independent proportions were calculated using the method of Agresti and Caffo (2000). Confidence intervals that exclude zero (highlighted in bold) provide evidence of a statistically significant change at the α = 0.05 level.

In contrast, at the expert stage, a significant transformation occurred. Among the 18 responses at expert stage (7 for unidirectional acceptance, 7 for negotiated revision, and 4 for position maintenance), the proportion of unidirectional acceptance dropped markedly from 70% to 39% while negotiated revision increased markedly from 5% to 39%. The 95% confidence intervals for these differences confirm their statistical significance (see Table 8). This transformation highlights Song’s evolution into an active participant in scholarly dialogue, engaging in negotiation with reviewers. Meanwhile, the proportion of stance maintenance remains stable, from 25% to 22%, showing there was no statistically significant change.

#### 4.2.2 Unable to detect the intentions of the reviewers at novice stage

Among the 5 maintain position responses of Paper 2007, 1 is about scholarly engagement and 4 are about technical depth. Song maintained his position in the 4 responses out of a total of 7 responses to technical depth comments, which is not common at novice stage. Let’s take a look at one of them.

The major concern of this reviewer is the lack of comparative results. The authors merely say that…. For a precise analysis, this reviewer would have expected at least a comparison with the classic … (which is missing in the figures). (Comment 1, Paper 2007)

This is the first comment from Reviewer 1. Obviously, the reviewer considered this to be a significant flaw of experimental design. However, Song without much experience of communicating with reviewers could not perceive this was a comment that should be revised. He maintained the position from his own perspective, explaining that the differences were not obvious and could not be distinguished in the same figure, and then explained why the differences were not obvious:

… After very careful investigation we find that the difference between … of the two approaches are so trivial that we nearly can not distinguish them in one figure. Therefor the comparison result is not included in the revised paper. Our explanation on this phenomenon is that … (Response to comment 1, Paper 2007)

Regarding this point, we checked the review comments of two other reviewers and found that reviewer 2 had similar suggestions to add performance comparison. However, Song did not take the same suggestion raised by two reviewers. In peer review, novice authors tend to fully accept or completely reject review comments, and are less likely to adopt a consultative response. This phenomenon reflects a stage characteristic in the process of academic socialization, that is, falling into a black-or-white dichotomous response pattern (Matsuda and Tardy, 2007).

Thus, in the second round of review, Reviewer 1 acknowledged author’s efforts to paper modifications, but specifically questioned for Song not providing comparison results as required:

The authors addressed the specific comments of this reviewer correctly. Mistakes related to mathematical formulae and inaccuracies in some explanations have been suitably fixed. This reviewer finds difficult to understand why the authors did not provide any comparative simulation results between … (Comment from reviewer 1, 2nd round, Paper 2007)
It is my opinion that the authors did not reply fully satisfactorily to the comments of the first review. In particular: My comment about the need to compare the presented approach with other approaches. (Comment from reviewer 2, 2nd round, Paper 2007)

Obviously, the novice Song could not detect that adding results of performance results is the main concern of two reviewers and it is a comment that must be adopted. Perhaps Song only focused on revising the article and failed to carefully understand the intentions of the reviewers. It can be seen that novice authors exhibit relatively weak academic social skills in interacting with reviewers. Fortunately, both reviewers were friendly and gave Song another chance to revise. The editor’s decision was being accepted with mandatory revision.

In this round of revision, Song added results and figures of performance comparison under various conditions following reviewers’ suggestion. During our interview, Song deliberately showed us with the added figures, explaining:

Look at these figures. We later used red dashed line and black straight line to represent the results of the classic algorithm and the new algorithm, respectively. You see, the red dashed line and black straight line almost overlap. This paper does not indicate how significant the results are. The paper only intended to demonstrate how fast the new algorithm runs and how small the computational load is, so we did not add the red dashed line in the first revised version. (Interview, May 2024)

When reviewing Song’s responses together with Song in this interview, Song once exclaimed: “My tone in the responses was so tough at that time.” Song admitted that he did not think too much about the risk of being rejected if he did not follow the reviewer’s comments at that time probably because he was young and inexperienced, and could not detect reviewers’ intention. Later, Song pays more attention to understanding the intentions of the reviewers and try to avoid situations where necessary revisions are not made. If his students encounter such review comments now, he will guide them to address the reviewers’ primary concern directly. It is meaningless to argue with reviewers with the risk of being rejected. Publishing papers earlier can help students defend and graduate earlier. Of course, from the perspective of the completeness of paper framework, adding performance comparison could be more comprehensive, Song eventually admitted to this during our interview.

#### 4.2.3 Persuading reviewers at expert stage

In Paper 2021, 4 position maintenance responses were almost evenly distributed in three dimensions of technical depth (1 response), scholarly engagement (2 responses), and text organization (1 response). 4 responses to technical depth comments out of a total of 7 underwent negotiated revision, which reflected the author’s growth in academic social skills after more than 10 years of interaction with peer reviewers.

As we have mentioned earlier, the comments from Reviewer 1of Paper 2021 were unexpected for Song. After carefully reading Reviewer 1’s overview of the new algorithm, Song found that Reviewer 1 probably did not understand this paper very well. By reading detailed comments raised by Reviewer 1, Song became more convinced of his finding. For example, one of Review 1’s comments is about a term used in the paper. The reviewer commented that there is no need to make up a term and the new term is confusing.

In this paper, the “AB” is confusing, I think it is only the change of the P phase to Q phase, should not be called as “AB.” You don’t have to make up a word. (Comment 5, Paper 2021)
Song explained in our interview:From this comment, it is very likely that Reviewer 1 is not from our field as the overview is not accurate and the reviewer is not very familiar with the terminology. This is very common. What we research is very technical and specific, and even if the reviewers are from the same specific field as ours, their research on this topic is probably not as deep as ours. In many of the cases, we can say authors know their own research best (Interview, May 2024).

In Song’s view, the major revision this time is to add clear explanations to the paper for audience not in this field to understand, as well as to respond seriously and carefully to the editor and Reviewer 1 in order to pass the second round of review.

Our response must emphasize the contribution of the paper while respecting the reviewers. The reviewer did not agree with this contribution, which is actually due to our unclear explanation in the article. In addition, regarding the issues raised by the reviewers, as long as there is reasonable aspect, it is necessary to admit the weaknesses before maintaining our position (Interview, May 2024).

Therefore, although Reviewer 1 of Paper 2021 did not fully agree with its publication, the author’s (Song’s) 18 responses were predominantly conciliatory: only 4 were maintaining position, the other 14 are negotiated revisions or unidirectional acceptances. Let’s take a look at two of negotiated responses.

For reviewer 1’s question about value of this paper, Song’s responses to the editor and Reviewer 1 are as follows:

Concerning the novelty doubted by reviewer 1, we have tried our best to illuminate it. We believe that from the viewpoint of scientific research, the significance and novelty of the paper lie in the following aspects: (1) …; (2) …; (3) … (Response letter to editor, Paper 2021)
The two convolution operations as described by the reviewer are portions of our paper. However, the significance and novelty of the paper lie in the following aspects: (1) …; (2) …; (3) … (Response to comment 1, Paper 2021)

In the above responses to comment 1, we can see that Song skillfully pointed out that the reason why Reviewer 1 does not agree with the contribution of this paper is probably the incomplete understanding: “The two convolution operations as described by the reviewer are portions of our paper.”

For Reviewer 1’s previous comment that Song does not have to make up a word, Song’s response is as follows:

We are very sorry to make such confusion here. As the reviewer has indicated, the AB is indeed the change of P phase to Q phase. However, we call it AB just following the classical paper by … (“Title of the paper” *Title of the Journal.*, vol. n, no. n, pp. n-n, Mar. 1996). They stated in this paper that. The following two figures are screenshots from this classical paper…
We regret that this confusion has greatly reduce the clearness of the rest of the paper. So we try to explain the origin of AB in the revised manuscript (the last paragraph in Page 5). Section IV has been revised to avoid such confusion, and the revisions have been highlighted.

This response is even more considerate, first stating that the reviewer did not know this term because the author did not explain it clearly, and then defending the author’s position of the existing term. From the above responses, we can see how an experienced author responds to the reviewer’s comments with his academic interaction competence. The review result of the second round was both expected and unexpected. What is expected is that Reviewer 1 acknowledged the author’s revisions and agreed to publish the paper. What is unexpected is that Reviewer 1 provided no other comments except the following one:

The authors have revised this manuscript carefully according to my comments and I think this paper could be accepted for publication. (Comment from Reviewer 1, 2nd round, Paper 2021)

It can be seen that Song’s careful revision and skillful responses have gained the recognition of Reviewer 1. In the second round of revision, Song mainly addressed some small issues such as formula derivation descriptions raised by Reviewer 2. Two months later, Song received an acceptance notice. But Song unexpectedly found that a Reviewer 3 had been added to review the paper. Obviously, the editor intended to hear one more voice. Here are the comments from three reviewers of this round:

This paper can be accepted. (Comment from Reviewer 1, 3rd round, Paper 2020)
I think this paper could be accepted for publication. (Comment from Reviewer 2, 3rd round, Paper 2020)
I found the paper well written and suitable for publication. It is clear, the manuscript has already been reviewed by other experts and I also appreciated the answers and final changes given by the authors in response. (Comment from Reviewer 3, 3rd round, Paper 2020)

Thus, Paper 2021, which accumulated Song’s 9 years of research achievements, was finally published. When asked if Song was worried that Reviewer 1 would stick to his original stance, Song replied:

In fact, the review process affords authors an opportunity to defend their work through. What we can do is to try our best to respond to the comments and revise the paper accordingly. As long as our responses are well-reasoned and respectful, the reviewer is unlikely to maintain the objection. Of course, if the manuscript is rejected, we can submit it to a different transaction (Interview, May 2024).

### 4.3 The changes in author academic participation

#### 4.3.1 Raising reputation by publishing SCI papers at novice stage

In 2005, Song had a need for promotion to professor. Although there were not strict requirement of publishing SCI papers for professional title promotion at that time, Song had an interest in giving it a try. In Song’s field, high-ranking SCI journals are IEEE transactions and letters, among which IEEE transactions with high impact factors are more influential. Song explained the reason for choosing ITX as his target journal:

ITX is prestigious and renowned with high impact factor in my field. At that time, I thought that if I intended to publish a SCI paper, it must be in a high-impact journal. And I knew through regular reading that ITX papers tend to be theoretical, that is, the proposed ideas may not necessarily be immediately implemented in the practical system due to the computational complexity, but they are interesting and significant. I just wanted to try my luck as my new algorithm fits. (Interview, Mar 2024)

As high-impact journals are frequently read and cited by peers, the publication of Paper 2007 brought academic reputation to Song and many peers got to know him through this paper. We can see that his position on the periphery of academic circle motivated his publication. His familiarities with the topics discussed at the center enabled him to find a voice, while the reviewers’ feedback solidified his understanding of what constitutes a high-quality paper. With these, Song started his international academic participation and gradually published many papers in IEEE transactions and letters on new progress of his scientific research. Soon with these SCI papers, Song was promoted to professor and gained reputation in this circle.

#### 4.3.2 Sharing research achievement at expert stage

The proposed algorithm in Paper 2007 is a small trick and was tested on computer simulations. The authors of the paper are only Song and his supervisor. However, Paper 2021 targets a relatively large and unresolved issue. More peers are involved in this engineering project from forming a two-step algorithm after repeated experimentation, to processing data after flight tests with collaboration with research institutes. Paper 2021 has seven authors including Song, young faculty members in his research team and his doctoral and master’s students. We can see that Song’s academic strength has been continuously promoted from following his supervisor to building his own research team and collaborating with research institutes for testing. In Song’s words:

Paper 2007 is written for professional title while Paper 2021 is for sharing scientific research achievements of years. (Interview, Oct 2024)

It can be seen that although international publication has earned Song reputation, with the enhancement of Song’s academic strength, collaborating with research institutes to complete practical engineering projects has become the next major goal. This is because:

First, the level of technological development varies in different countries, so do the topics discussed in the paper publishing circle. Technologies that are mature in some countries may need constant improvement in other countries. Or technologies that are not used in some countries may be widely applied in other countries. What’s more, in the field of engineering, the main goal of scientific research is to solve practical problems. Not all problems are written in papers after they are solved. Some countries do not have a tradition of publishing papers probably for the sake of confidentiality or others. In other countries who do, it is mainly the researchers in research institutes who publish papers or students and young faculty members in universities who have the temporary demand of graduation or title promotion. That is to say, there are technologies that have not been written into papers and there are researchers and faculty members who do not write papers (Interview, Oct. 2024).

So the paper publishing circle is incomplete, at least in the field of engineering. International publishing help boost one’s reputation in a circle only involving those who have the tradition to publish or have the demand to publish. Such international participation is meaningful but also limited to a certain group of peers. Therefore, it is more appropriate to say that international publishing and peer interaction helps one move from periphery to center of a paper publishing circle, not the academic circle. Due to the incomplete academic circle, researchers like Song will shift their focus to completing engineering projects after realizing their promotion of professional titles. Occasionally, they write papers to record and share their latest research findings.

## 5 Discussion

This study reveals the impact of peer review interaction on author’s academic growth through a mixed research approach. The research findings provide diachronic evidence for the study on the path of academic socialization.

### 5.1 Paper quality changes and writing competence development

Despite the lack of quantitative changes in comment classification, qualitative evidence from the author’s reflection on the novice-stage paper reveals that the review process led to a profound reconceptualization of international journal standards. This qualitative shift, which triggered significant manuscript revisions, ultimately contributed to the well-constructed expert-stage paper, confirming academic writing development stages (Belcher, 2007). This shift reveals an important mechanism: through continuous reviews and responses, expert authors gradually internalize disciplinary standards, developing anticipatory writing competence that allows them to predict and address potential review concerns during the drafting process, thereby demonstrating genre cognitive development (Tardy, 2016; Liu and Chen, 2022).

### 5.2 Social skill changes and academic identity construction

In terms of the development of academic social skills, the phenomenon of unidirectional acceptance of review comments during the novice stage echoes the discovery of the initial passivity of novice authors (Li and Flowerdew, 2020; Darvin and Norton, 2019; Tian and Liu, 2024). The negotiated behavior during the expert stage reflects the awakening of rhetorical agency. This transformation breaks through the unidirectional model of socialization in peer review (Flowerdew, 2000), revealing the bidirectional nature of academic identity construction: expert authors not only response comments, but also maintain their position through strategic responses (such as providing literature evidence and clarifying paper novelty). This further echoes Hyland’s (2015) theory of academic interaction rituals: the review process is not only a means of quality control, but also a key field for scholars to engage in academic community from standard followers to standard co-builders.

### 5.3 Academic participation changes and academic accumulation

In terms of academic participation, we can see the change from paper publishing at novice stage to engineering project research and implementation at expert stage. Peer review interaction is also a key reflection for the transformation of academic participation motivation. When novice authors are driven by external factors such as promotion of professional titles, they often simplified peer review as a task-based behavior to avoid controversy and maintain safe publication. With the increasing of academic accumulation, expert authors have the responsibility to share their recent work driven by intrinsic motivation (Deci and Ryan, 1985; Li et al., 2025). They regard peer view interaction as a means to maintain discipline standards. This transformation challenges the single channel for academic guidance (Casanave, 2003), demonstrating that the peer review interaction itself constitutes an important field for academic participation transformation.

## 6 Conclusion

This longitudinal case study systematically traces paper writing, review, and revision trajectory of one author spanning 14 years. Peer review triggers authors’ academic growth through: the internalization of paper standards sparks a shift from mechanical imitation to intentional construction, the practice of responding to comments promotes a shift from unidirectional acceptance to negotiated revision, and the change in academic participation undergoes a shift from external regulation to intrinsic motivation. These findings empirically validate the co-constructive nature of academic publication, offering pedagogical strategies that bridge paper drafting with invisible academic socialization and open up a dynamic analysis path for the research of occluded genres.

### 6.1 Consideration of alternative explanations and boundaries

It is crucial to acknowledge the boundaries of these interpretations. As a single-case study situated in a rapid-iteration engineering discipline where technical feasibility is often prioritized, the generalizability of findings to theoretical or humanities-oriented fields may be limited. Furthermore, we have considered alternative explanations for the observed growth. While factors like career advancement, expansion of academic networks, or the learning of submission strategies undoubtedly play a role in this study, the documented textual interactions provide strong evidence that the peer review interaction itself was a core and direct catalyst for the identified shift. Meanwhile, the author’s leading role in two papers and the consistency of the targeted journal authority help mitigate confounding effects from team expansion or vastly differing editorial alleviate the confusion caused by team expansions or vastly different editorial expectations. Therefore, the tripartite mechanism is a robust but contextualized explanation derived from the data.

### 6.2 Implications and further research

Based on the above, this study proposes three curriculum suggestions for academic English writing. The first involves a staged training framework. The foundational curriculum applies models such as Swales’ (1990) CARS (create a research space) to apply learners’ understanding of paper quality criteria in manuscript writing. Advanced courses require the establishment of rhetorical negotiation simulation to decode reviewers’ comments and cultivate response strategies, such as addressing comments by detecting the intention of reviewers. Secondly, drawing on situational learning theory (Lave and Wenger, 1991), a “peer review community” practical training project can be constructed. This project can introduce a phased training model similar to the “reviewer residency” (Munasinghe et al., 2022) allowing learners to deeply experience review interaction and negotiation through the progression of roles from co-review, supervised independent review and unsupervised independent review, thus initiating their academic socialization process. The third is the establishment of a dynamic evaluation system. The system can include response strategies and discourse ownership of occluded genres, moving away from traditional outcome-oriented assessments. By emphasizing process-oriented and context-sensitive criteria, the evaluation system can better align with the complexities of academic writing.

Future research can be studies in the following directions: firstly, it can be extended to interdisciplinary comparative research to explore the similarities and differences in peer review impact on the academic growth of scholars among natural sciences, engineering, humanities and social sciences. The essential competence of academic socialization is more than just interacting with reviewers. Secondly, it is suggested to introduce a cultural sociology perspective to compare the cultural differences in academic communities of different countries. What’s more, it is recommended to pay attention to the reconstruction effect of emerging technologies such as preprint platforms and AI assisted review on traditional occluded genres and review mechanisms.

## Data Availability

The raw data supporting the conclusions of this article will be made available by the authors, without undue reservation.

## Footnotes

1 IEEE transaction papers have the following review results: publish unaltered (A), publish in minor, required changes (AQ), review again after major changes (RQ) and Reject (R).
